# Gene expression profiling of human mesenchymal stem cells derived from bone marrow during expansion and osteoblast differentiation

**DOI:** 10.1186/1471-2164-8-70

**Published:** 2007-03-12

**Authors:** Birgit Kulterer, Gerald Friedl, Anita Jandrositz, Fatima Sanchez-Cabo, Andreas Prokesch, Christine Paar, Marcel Scheideler, Reinhard Windhager, Karl-Heinz Preisegger, Zlatko Trajanoski

**Affiliations:** 1 Institute for Genomics and Bioinformatics and Christian-Doppler Laboratory for Genomics and Bioinformatics, Graz University of Technology, Graz, Austria; 2 Department of Orthopaedics, Medical University of Graz, Graz, Austria; 3 Eccocell Biotechnology Inc., Graz, Austria

## Abstract

**Background:**

Human mesenchymal stem cells (MSC) with the capacity to differentiate into osteoblasts provide potential for the development of novel treatment strategies, such as improved healing of large bone defects. However, their low frequency in bone marrow necessitate *ex vivo *expansion for further clinical application. In this study we asked if MSC are developing in an aberrant or unwanted way during *ex vivo *long-term cultivation and if artificial cultivation conditions exert any influence on their stem cell maintenance. To address this question we first developed human oligonucleotide microarrays with 30.000 elements and then performed large-scale expression profiling of long-term expanded MSC and MSC during differentiation into osteoblasts.

**Results:**

The results showed that MSC did not alter their osteogenic differentiation capacity, surface marker profile, and the expression profiles of MSC during expansion. Microarray analysis of MSC during osteogenic differentiation identified three candidate genes for further examination and functional analysis: ID4, CRYAB, and SORT1. Additionally, we were able to reconstruct the three developmental phases during osteoblast differentiation: proliferation, matrix maturation, and mineralization, and illustrate the activation of the SMAD signaling pathways by TGF-β2 and BMPs.

**Conclusion:**

With a variety of assays we could show that MSC represent a cell population which can be expanded for therapeutic applications.

## Background

In recent years mesenchymal stem cells (MSC) have generated a great deal of interest as a potential source for cell-based therapeutic strategies. Human MSC are easy to isolate from small aspirate of bone marrow via their adherence ability. These cells readily generate single-cell-derived colonies that can be highly expanded and differentiated into a variety of cell types, such as osteoblasts [1,2], adipocytes [3], myocytes [4], astrocytes and neurons [5,6]. Further, human MSC can improve cardiac function after infarction [7,8] or symptoms of bone and cartilage defects [9-13], as well as neurodegenerative diseases such as Alzheimer's [14-16]. Their efficiency in multiple types of cellular therapeutic strategies has been demonstrated, including applications in treating children with *osteogenesis imperfecta *[17], hematopoietic recovery [18], and bone tissue regeneration [19,20]. Also first preclinical trails are in progress to test their capacity and toxicity in applications for human treatment [21].

One great advantage of MSC is that these cells may be directly obtained from individual patients, thereby eliminating the complications associated with immune rejection of allogenic tissue and infectious diseases. However, for cell therapies MSC have to be expanded and/or manipulated to obtain a sufficient amount of cells that can be subsequently used for treatment. Despite growing experience and knowledge concerning human MSC and their use in cell-based strategies, the molecular mechanisms that govern MSC self-renewal, expansion and multilineage differentiation are not well understood and remain an active area of investigation.

In this study we asked if human MSC are developing in an aberrant or unwanted way during *ex vivo *long-term cultivation and if cultivation conditions exert any influence on their stem cell maintenance. To address this question systematically and comprehensively we first developed human oligonucleotide microarrays with 30.000 elements and then performed large-scale expression profiling of long-term expanded MSC isolated from clinically relevant samples. We monitored these cells during their expansion *ex vivo *with respect to proliferation kinetics, surface marker profile and differentiation potential. Finally we analyzed the gene expression profiles of MSC during osteogenic differentiation. Our results showed that expansion of MSC does not result in substantial genetic and morphological aberrations. We illustrated for the first time in a human model the three main stages of osteogenic development, and we could show the diverse regulation of the SMAD pathways by TGF-β2 and BMPs.

## Results

### Human MSC maintain their undifferentiated phenotype during long-term expansion

The results of the *ex vivo *long-term expansion experiments showed that the undifferentiated phenotype of MSCs is maintained with respect to differentiation potential, surface marker profile and gene expression profiles. At confluence of 75–85% the cultivated cells were detached and flow cytometry analysis were performed to verify the purity of the cell population without any contaminations, such as haematopoietic cells. Following surface marker profile were detected: CD44+, CD90+, CD73+, CD105+, CD166+, CD11b-, CD34-, CD45-, CD117-, HLA DR-. For the subsequent experiments MSCs were expanded until the end of the tenth passage. At the end of each passage the cells were analyzed by flow cytometry and a cell aliquot was seeded out for testing their differentiation ability.

#### Proliferation kinetics and differentiation potential

**Figure 1:**
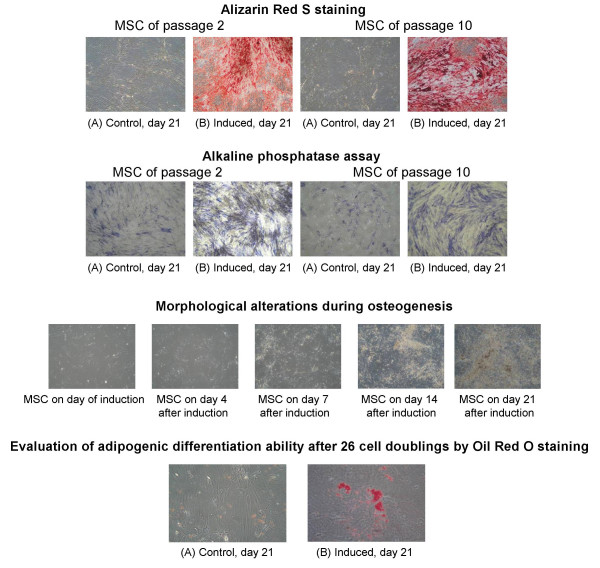
**Upper panel: **Osteogenic differentiation of MSC after passage 2 (A, B) and passage 10 (C, D). Alizarin Red S staining and Alkaline phosphatase assay were performed after 21 days of osteogenic induction (d21).100× magnification. **Middle Panel: **Morphological alterations during osteogenesis determined by microscopy on the day of induction (d0), day 4, 7, 14 and 21 after induction. All pictures are shown in 100× magnification. **Lower Panel: **Adipogenic differentiation of MSC in passage 10 characterized by Oil red O staining; (A) MSC not induced, undifferentiated; (B) MSC 21 days after induction with adipogenic medium 100× magnification.

The growth kinetics of five donors was investigated from the primary culture through the tenth passage, corresponding to approximately 26 cell doublings. For recalculating the starting number of MSC in the MNC fraction, CFU assays were performed and the MSC frequency was determined. Primary cultures reached their first confluence from around 80% in about 2 weeks and about 10 cell doublings. During the following passages the proliferation rate slowed down. During the whole expansion period – starting with passage two until passage ten – in every passage the osteogenic differentiation ability was tested. The osteogenic differentiation was confirmed by Alizarin Red S staining and Alkaline Phosphatase assay. Throughout this examination period differentiation ability into osteoblasts was observed in low passages (passage 2, after 12 cell doublings) as well as in a higher passage (passage 10, after 26 cell doublings), (Figure 1). MSC expanded to approximately 26 cell doublings were able to differentiate into adipocytes (Figure 1).

#### Surface marker profile

After the cultivation of MSC from the mononuclear cell fraction (passage 0) and at the end of each individual passage, the MSC were analyzed by flow cytometry to monitor the surface marker profile during their *ex vivo *expansion. Each investigated passage depicted homogenous cell population demonstrating that MSC did not alter their physical and morphological properties during high grade *ex vivo *expansion under the culture conditions tested [see Additional file 1]. In addition to the examination of the surface marker profile, we also performed Real-time RT-PCR Sybr™ Green I assays for stem cell specific genes. The examined stem cell markers, CD44, CD73, CD166, CD105, CD90 and STRO1 showed stable expression level during the whole expansion period proving the results obtained by FACS analyses. As expected, CD34, CD45 and TERT could not be detected on any examined time point [see Additional file 2].

#### Large-scale gene expression profiling of MSC during *ex vivo *expansion

In order to monitor the molecular events that govern the *ex vivo *expansion of undifferentiated MSC we performed large-scale gene expression profiling using oligonucleotide microarrays with 29952 elements (ArrayExpress, accession numbers A-MARS-2, E-MARS-5, and E-MARS-6, [see Additional file 3, 4, 5]). After background correction, global mean and dye swap normalization, and replicate handling, 838 genes could be detected as differentially expressed with at least 2-fold change, between cells from passage 2 with cells from passage 5, and 345 genes, when comparing passage 5 cells with passage 10 cells [see Additional file 6]. A modified version of the regularized *t*-test [22] with the complete data set was performed to identify significantly differentially expressed genes in ten examined donors (Nr. 1–10, passage 2 vs. passage 5) and five examined donors (Nr. 6–10, passage 5 vs. passage 10). The results showed that only 9 genes were significantly differentially expressed (p < 0.05) during 8 cell doublings when comparing cells from passage 2 versus cells from passage 5 (Table 1). Significantly differentially expressed genes represent proteins with diverse functions: extracellular matrix proteins (Col11A1, tenascin xb), proteins involved in bone, fat and epithelial cell metabolism (osteoprotegerin, b219ob receptor, FGF- 7), pain perception and stress response (proenkephalin), growth-promoting activity (IGF-2), and signaling and cell survival mechanisms (semaphorin 3c). The hypothetical protein XP_016240 is annotated as similar to keratinocyte growth factor-like protein, and the hypothetical protein dkfz434b044 is defined as a cysteine-rich secretory protein containing LCCL domain 2. The expression data from the above mentioned genes could be confirmed by independent real-time RT-PCR [see Additional file 7].

**Table 1:** Differentially expressed genes of MSC after high grade expansion.

**Passage 2 vs passage 5 Acc.No**	**Gene name**	**log2 ratio**	**Fold-change**
XM_016240	hypothetical protein xp_016240; loc87477//Homo sapiens similar to keratinocyte growth factor-like protein, group II – human (LOC158116)	0.92	1.89
NM_006211_1	proenkephalin; penk	1.58	2.99
NM_001854_1	collagen, type xi, alpha 1; col11a1	1.27	2.41
BC010956_1	similar to fibroblast growth factor 7 (keratinocyte growth factor)	1.03	2.04
NM_000612_1	Insulin-like growth factor 2 (somatomedin A); igf2	1.59	3.01
NM_031476_1	hypothetical protein dkfzp434b044; dkfzp434b044	1.34	2.53
U52914_1	b219ob receptor isoform hub219.3 precursor	1.13	2.19
NM_006379_1	sema domain, immunoglobulin domain (ig), short basic domain, secreted, (semaphorin) 3c; sema3c	-0.69	0.62
NM_002546_1	osteoprotegerin; tnfrsf11b	-1.03	0.49
**Passage 5 vs passage 10 Acc.No**			
NM_019105_1	tenascin xb	0.85	1.80

List of differentially expressed genes after statistical analysis by modified version of the regularized t-test [64] (p < 0.05) for ten donors (passage 2 vs. passage 5) and five donors (passage 5 vs. passage 10).

In sum, these results show that long-term expansion of MSC did not alter their differentiation potential, surface marker profiles, and expression profiles.

### Expression profiling of MSC during osteogenic differentiation reveals distinct phases of osteogenic development

#### Osteogenic differentiation

Human MSC from three different donors (Nr. 9–11) were cultured until their fourth passage and were induced to osteogenic differentiation at a confluence of 70–80%. The osteogenic differentiation was observed during the whole period by microscopy and was terminated on day 21 after induction. First signs for calcification appeared as black regions within the cell monolayer after around seven days. The maximum of calcified extracellular matrix was observed after 21 days of treatment (Figure 1). The osteoblast phenotype was confirmed by Alkaline Phosphatase assay and Alizarin Red S staining (data not shown).

#### Expression of osteoblast specific marker genes

We performed Real-time RT-PCR assays to detect the expression levels of osteoblast specific marker genes MSX2 (homeobox gene MSX2), VDR (vitamin D receptor), COL1A1 (collagen 1A1), ALPL (alkaline phosphatase), SPARC (osteonectin), SPP1 (osteopontin) and BGLAP (osteocalcin) to prove the osteoblast phenotype. The log2 transformed results are shown in the supplementary material [see Additional file 8].

The data obtained by Real-time RT-PCR showed the expected expression profiles of the osteoblast phenotype. ALPL showed the expected expression level progression. Its expression was increased starting with day four of the differentiation period and decreased after day 14 until day 21 during the mineralization phase. The homeobox gene MSX2, which is implicated in osteoprogenitor cell function [23] and an up-stream regulator of RUNX2, which was described to be important for the osteogenic differentiation [24], were up-regulated during the whole period of differentiation. COL1A1 is known to be an early marker of osteoprogenitor cells [25]. Its maximum of expression was reached on day 21. Also SPARC showed a nearly constant, up-regulated expression level with its maximum on day 21 of differentiation. SPP1 is an extracellular matrix protein, known to peak twice in its expression [26]: around day 4, during proliferation, and between days 14–21, during mineralization. BGLAP was described as a late marker of developing osteoblasts appearing with matrix mineralization [27] and was maximally expressed on day 21. Interestingly, BGLAP was already significantly up-regulated on day four, whereas the mineralization could not be observed before day seven.

#### Large-scale gene expression profiling of MSC during osteogenesis

The RNA was harvested at four time points during the differentiation period and hybridized on human oligonucleotide microarrays. As reference, RNA harvested from MSC one day before the osteogenic induction was used (ArrayExpress, accession numbers A-MARS-2 and E-MARS-3). After LOWESS-subgrid normalization and filtering missing values 1108 genes were selected with two-fold up- or downregulated expression in at least one time point [see Additional file 9].

#### Correspondence between co-expressed genes and phenotypic changes

**Figure 2:**
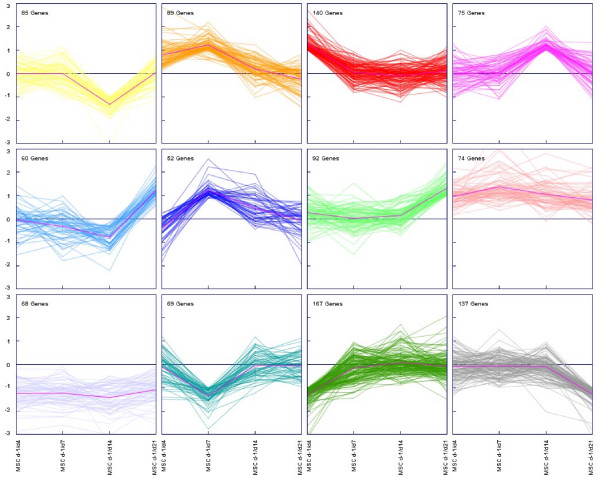
Expression view of all clusters calculated by *k-*means clustering. *K*-means clustering was performed for 1108 selected genes/ESTs shown to be more than two fold up or down regulated in at least one time point during osteogenic differentiation. Genes were grouped in 12 clusters with distinct expression profiles. Relative expression levels (log2 ratios) are shown for each gene at different time points and for the mean expression values (magenta line).

Differentially expressed genes were clustered according to their expression profiles using the *k-*means clustering method [28] [see Additional file 10]. The cluster analysis was performed with at maximum 50 iterations and using 12 different clusters, each containing between 58 and 167 genes (Figure 2). The number of clusters was estimated by FOM analyses [see Additional file 11]. Additionally, hierarchical clustering was performed and the results were comparable to *k*-means clustering [see Additional file 12].

**Figure 3:**
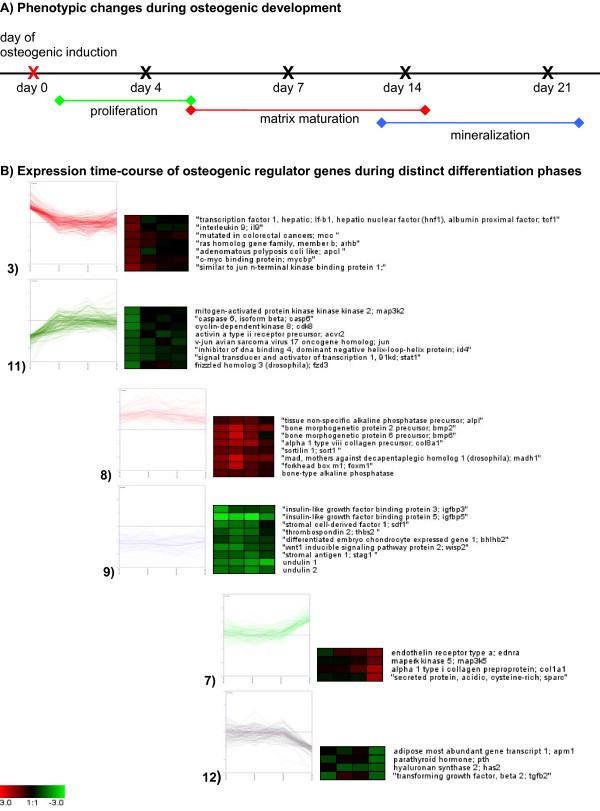
Summary of osteogenic development; a) Graphical summary of the 3 phases of osteogenic development merged with the time scale; b) Chosen clusters after *k*-means clustering describing each developmental phase merged with the expression matrix of distinct key regulators.

By analyzing the genes of the different clusters and their expression profiles, we were able to reconstruct three main phases of osteogenic development (Figure 3): proliferation, matrix maturation, and mineralization. Additionally, novel candidate genes could be found which are not known to play a role during osteogenic differentiation: ID4 (inhibitor of DNA-binding), CRYAB (crystalline-αB) and SORT1 (sortillin1). The expression of these genes was subsequently confirmed by quantitative RT-PCR [see Additional file 13].

**Figure 4:**
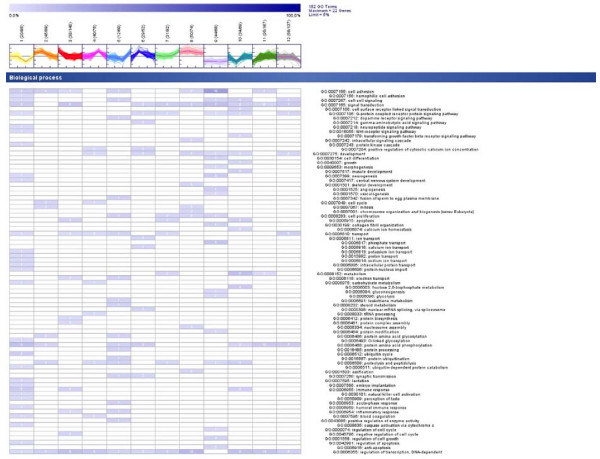
Distribution of gene ontology (GO) terms regarding biological processes for genes in each cluster. The GO terms listed here are those present in at least 5% of the genes within the cluster. In brackets are the number of genes with associated GO terms and the number of genes within the cluster.

For gene ontology (GO) analysis we categorized ESTs with available RefSeq annotation (506 RefSeq annotations out of 1108 differentially expressed genes) according to GO terms for biological process (Figure 4), cellular component, and molecular function [see Additional file 14]. In detail, cluster 3 and 11 represented proliferation phase describing genes, which were involved in signal transduction, regulation of cell cycle, metabolism and regulation of transcription. Cluster 8 and 9 illustrated genes which were up-or down regulated during the whole period of differentiation. They describe the phase matrix maturation and are involved in development, cell differentiation, metabolism, regulation of DNA-dependent transcription. The phase of mineralization is evident in cluster 7 and 12, which include genes involved in signal transduction, transport, metabolism, and development.

The GO analysis showed that genes found in cluster 2 and 6 were also involved in cell adhesion, signal transduction, cell cycle, and regulation of transcription, similar to cluster 3 and 11. Cluster 10 described genes of development and metabolism among the rest. Cluster 1 and 5 combined genes of cell adhesion, transport, cell proliferation and regulation of transcription. Cluster 4 represented genes associated with transport and regulation of transcription.

In summary, GO annotation showed that nearly in each cluster genes of cell adhesion, signal transduction, transport, and regulation of transcription were present, illustrating the complex regulation of different events during osteogenesis.

#### Pathway analysis of the expression data set

**Figure 5:**
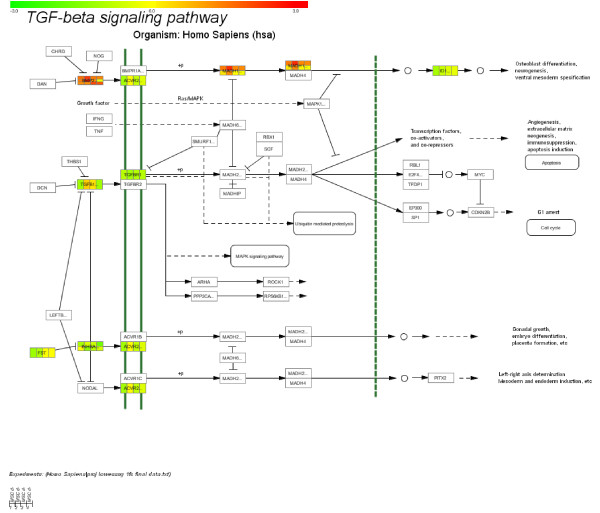
Illustration of the TGF-β signaling pathway derived from KEGG database with mapped genes according to their expression level (at least two fold up or down regulated); green = down-regulated genes, red = up-regulated genes and yellow = unregulated genes; mapped genes are at least two-fold differentially expressed.

The data set of 1108 genes with significant up or down regulation (+/- 2 fold, p ≤ 0.05) was mapped with the Pathway Explorer on pathways derived from the Biocarta and KEGG database [29,30]. In total 276 out of 1108 genes could be mapped with their available RefSeq numbers (24.9%). Only pathways where at least 10% and more than five mapped genes could be found were selected for further examination. Following these criteria the TGF-β signaling pathway (12 mapped genes out of 116, 10.38%, see Figure 5) was ranked first, which summarized the different Smad pathways regulated by the members of the TGF-β family: BMP, TGF-β and inhibin.

Cell growth and proliferation is controlled by the members of the TGF-β superfamily which includes structurally related proteins like TGF-β, activins, and bone morphogenetic proteins (BMPs) [31-34]. In the here presented TGF-β signaling pathway BMP2 and BMP6, MADH1 and MADH9 (corresponding to SMAD1 and SMAD9) were up-regulated and the inhibitor of DNA binding ID4 was down-regulated (Figure 5). BMP2 and BMP6 are able to bind to BMPR-type I and II, whereas BMP6 is able to bind to ActRIIA [35,36]. Due to the binding of these two molecules to the heterodimeric receptor complex the SMAD signaling pathway is activated. Our data illustrated further, that the inhibitor of DNA binding, ID4 was down regulated and the osteogenic differentiation could be induced. In parallel, TGF-β2, inhibin A and B were down-regulated during the differentiation period, whereby the following events were suppressed. Hence, under these experimental conditions the Smad-signaling pathway activated by BMP2 was responsible for the osteogenic development. In contrast, the activation of the Smad pathway by TGF-β and inhibin was suppressed.

### *In vivo *relevance of human MSC as *in vitro *differentiation model

The *in vitro *differentiation of MSC into osteoblasts is often used model to examine the regulation mechanisms during osteoblast development. To evaluate the *in vivo *relevance of this model, we compared the RNA obtained at the diverse time points during osteogenic differentiation with RNA isolated from primary cultures of normal non-mineralized human osteoblasts derived from hipbone using our microarrays (ArrayExpress, accession numbers A-MARS-2, and E-MARS-4) [see Additional files 15, 16, 17]. The results showed that MSC on day seven of their differentiation were most similar to human osteoblasts [see Additional file 18]. This observation could be confirmed by the comparison of the morphology of the two cell types: Human osteoblasts isolated from hipbone represented non-mineralized osteoblasts with fibroblast-like phenotype, morphologically similar to induced MSC on day seven after induction.

## Discussion

In this study we could show for the first time that MSC maintain their undifferentiated phenotype after *ex vivo *long-term expansion. The results of the microarray assays showed that MSCs did not change significantly their gene expression profiles during long-term expansion since only nine out of almost 30.000 genes were differentially expressed between passage 2 and passage 5 in ten donors. In addition, no changes in the expression profiles after additional 5 passages could be observed in five of the ten donors. These surprising results can only be partially explained by the limitations of the microarray technology. Due to the lower sensitivity and uncomplete coverage of the transcriptome, some genes required for stem cell maintenance might have been missed. Additionally, individual differences between the donors and slightly different conditions during tissue extraction and sample treatment could increase the number identified genes which were otherwise filtered out during averaging. However, the microarray results were supported by the analyses of the effects of extensive long-term *ex vivo *cultivation of MSC on their proliferation capacity, their morphology, surface marker profile, and differentiation ability. During the long cultivation time we could observe, in concordance with other groups [37,38], a progressive lost of proliferation ability and a slight shift in the cell-morphology of MSC of higher passage. However, their surface marker profile determined by flow cytometry (CD44+, CD90+, CD73+, CD105+, CD166+, CD45-, CD117-, HLA DR) and by Real-time RT-PCR was not altered. Over and above, the stem cell markers like CD90 and STRO1 showed stable expression levels, which further supports our observation that MSC maintained their undifferentiated phenotype and remained capable of osteogenic differentiation during *ex vivo *expansion at all tested passages (p2-p10).

Additionally to this clinically relevant evidence, we provide novel biological insights derived from the global view of the molecular processes during osteogenic development. First, we were able to illustrate the different phases of osteogenic development in a human model. Temporal expression of cell growth and osteoblast phenotype-related genes in a rodent model were first described by Stein et al. [39]. In our study, using large-scale expression profiling of clinical samples we could associate gene clusters to the three distinct phases of the development of osteoblast phenotype: proliferation, matrix maturation and mineralization.

Second, we could identify markers of late osteogenesis: ID4, CRYAB and SORT1:

a) ID4 is one of the four members of the ID (inhibitor of DNA binding) transcription factor family, which belongs to the basic-helix-loop-helix (bHLH) family. The ID members play a key role in differentiation processes and are involved in cell cycle control and cellular senescence [40-43]. It has been shown that ID proteins, by heterodimerization with bHLH proteins (MyoD, E12, E47), inhibit their binding to DNA, thus acting as dominant negative regulators, and have to be down-regulated to continue the differentiation process [44,45]. To the best of our knowledge only ID1, ID2 and ID3 have been identified as most significantly up-regulated early targets of the osteogenic BMP2, BMP6 and BMP8 [46]. In our study we provide evidence that also ID4 exhibits a specific role during the osteogenic differentiation.

b) The second identified gene is crystallin-αB (CRYAB). Alpha crystallins are small heat shock proteins and composed of two gene products: alpha-A and alpha-B, for acidic and basic. Alpha-A is preferentially restricted to the vertebrate eye lens where it maintains the transparency and refractive index of the lens, whereas alpha-B is widely expressed in many tissues and organs [47]. The here observed up-regulation of CRYAB suggests an involvement in osteogenic differentiation but its specific role is still unknown. This involvement is supported by Furushima et al. [48], who performed a linkage study with knowledge-based candidate genes to detect genetic determinants associated with OPLL (ossification of the posterior longitudinal ligament of the spine), a predominant myelopathy among Japanese. The candidate genes for examination were obtained by cDNA microarray analysis of gene expression profiles during osteoblastic differentiation of MSC. Among the 24 genes identified in cDNA microarray analysis which could be associated with bone metabolism, CRYAB was the only gene which showed significant evidence of linkage.

c) Further, we could show the role of sortilin1 during osteogenic differentiation. Sortilin 1 (SORT1) represents a multi-ligand type-1 receptor which binds a number of unrelated ligands that participate in a wide range of cellular processes. The expression of SORT1 was increasing until day seven and then slowly decreasing. Sortilin1, also known as neurotensin receptor-3, is a glycoprotein originally purified from human brain [49]. The expression of sortilin1 is ubiquitous; it has been observed in heart, brain, placenta, lung, skeletal, muscle, pancreas, prostate, testis, small intestine, thyroid, and spinal cord, whereas its expression in bone marrow could not be detected [50]. The function of srotilin1 in bone metabolism is poorly understood. Maeda et al. [51] reported first differential expression during osteogenic differentiation and performed further experiments, which showed that sortilin 1 is promoting extracellular matrix maturation. Our results presented here strengthen this hypothesis, since sortilin 1 shows a similar expression progression as other well known osteogenic genes, such as ALPL or BMPs.

In summary, we have identified by expression profiling and confirmed by quantitative RT-PCR three markers of late osteogenesis. Further studies are necessary to quantify the expression of ID4, SORT-1 and CRYAB in primary human osteoblasts and other cell types and to identify the role of these genes in osteogenesis.

Third, by mapping the obtained expression data on curated biomolecular pathways, we were able to illustrate the distinct activation of the different Smad pathways induced by BMPs, TGF-β and inhibin, which were summarized as TGF-β pathways. The mapping of the expression data demonstrated that the fate of cell differentiation was simultaneously controlled at three different points: 1) BMP2, BMP6 and BMP8 activated the Smad-signaling pathway, consisting of the main components Smad 1, Smad 5 and Smad 8 resulting in osteoblast differentiation; 2) TGF-β2, regulating Smad 2 and Smad 4, was down regulated and the subsequent mechanisms were suppressed; and 3) inhibin was, like TGF-β2, down regulated. These findings have been confirmed by recent reports describing the diverse Smad-signaling pathways, but have never been illustrated in a general overview before this study [52-55].

Finally, the comparison of *in vitro *differentiated osteoblasts with *in vivo *developed osteoblasts on morphological and genetic level proved the usefulness of MSC as *in vitro *model for the investigation of the osteogenic development.

## Conclusion

The here presented results demonstrate that MSC represent a cell population which can be expanded *ex vivo *and differentiated into osteoblasts. The genomic approach presented here represents a powerful tool to systematically and comprehensively investigate therapeutic approaches and hence, facilitate translational research on stem cells. However, it should be pointed out that new quality and safety standards have to be developed to guarantee risk free usage of *ex vivo *manipulated cell material for future therapeutic applications.

## Methods

### Cultivation and *ex vivo *expansion of human bone marrow-derived MSC and normal human osteoblasts

The mononuclear cell fractions (MNC) were derived from bone marrow from eleven different donors (Table 2), who gave consent after full information and approval by the hospital ethical committee (No. 12–091). All MNC were isolated from bone marrow aspirates at iliac crest during surgery. MNC were seeded at a density of 1 × 10^5 ^per cm^2 ^and cultured for 24 hours. After these 24 hours non-adherent cells were removed by medium exchange. The adherent cells were cultured in expansion medium containing DMEM (Invitrogen), 10% FBS (selected lot, Stem Cell Technologies), 100 U/ml penicillin, 100 μg/ml streptomycin and 2 mM L-glutamine under a humidified atmosphere of 5% CO_2 _at 37°C. Medium was changed three times a week. After reaching a confluence of 75–85% the cells were detached with 0.05% trypsin/1 mM EDTA and replated at density of 4 × 10^3^cells/cm^2^. The cells were expanded until the end of the tenth passage (corresponding to 26 cell doublings).

**Table 2:** Clinical characteristics of the donors.

**No.**	**Sex**	**Age**	**Clinical diagnosis**	**Therapy**
1	f	64.1	Arthrosis of hip joint	Total endoprothesis
2	f	49.5	Arthrosis of hip joint	Total endoprothesis
3	f	56.0	Osteoporotic fracture	Osteosynthesis
4	f	79.3	Arthrosis of hip joint	Total endoprothesis
5	m	78.9	Avascular necrosis of femoral head	Total endoprothesis
6	m	46.1	Avascular necrosis of femoral head	Total endoprothesis
7	m	81.6	Arthrosis of hip joint	Total endoprothesis
8	f	72.6	Arthrosis of knee joint	Total endoprothesis
9	m	38.4	Arthrosis of hip joint	Total endoprothesis
10	m	39.8	Arthrosis of hip joint	Total endoprothesis
11	f	79.5	Arthrosis of knee joint	Total endoprothesis

Sex, age, clinical diagnosis and therapy of the donors whose cells were used in this study.

The cells for the cultivation of normal human osteoblasts derived from hipbone were obtained by PromoCell (Heidelberg, Germany). The cryopreserved normal human osteoblasts were thawed and cultivated until the end of the fourth passage following the protocols and media recommended by PromoCell.

### Colony-forming-units of fibroblasts (CFU-F) assay

For determining the starting number of MSC in the mononuclear cell fraction CFU-F assays were performed. MNC from at least three donors were seeded in three concentrations, 2*10^6^, 1*10^6 ^and 0.5*10^6 ^cells per T25 flasks, and incubated for 14 days in expansion medium without medium exchange under a humidified atmosphere of 5% CO_2 _at 37°C. After 14 days the cell suspension was removed, the adherent cells were fixed with methanol, dried and stained for 5 minutes with Giemsa staining solution (VWR). After washing with distilled water the colonies were counted and the human MSC frequency in the MNC fraction was calculated.

### *Ex vivo *differentiation of human bone marrow-derived MSC in osteoblasts and adipocytes

For the induction of osteogenesis MSC were seeded at a density of 10^4 ^cells/cm^2 ^in expansion medium. After 24 hours the differentiation was induced by medium exchange. The osteogenic differentiation medium was based on the expansion medium supplemented with 10 nM dexamethasone, 0.1 mM ascorbic-acid-2-phosphate and 10 mM β-glycerophosphate. The *adipogenic *differentiation medium contained in addition to the expansion medium 0.1 μM dexamethasone, 50 μM indomethacin and 5 μg/ml insulin. The media were changed three times a week. Osteogenic differentiation was detected by Alkaline Phosphatase assay (Sigma) and Alizarin Red S staining of mineralized matrix, whereas adipogenic differentiation was detected by Oil Red O staining of the adipocyte specific fat vacuoles.

The *Alkaline Phosphatase assay *was performed with the Alkaline Phosphatase kit No. 85 purchased by Sigma following the manufacturers' instructions. For the *Alizarin Red S staining *a 1% Alizarin Red S in 2% EtOH staining solution was prepared. The medium was removed; the cultures were washed twice with preheated 37°C PBS, fixed with 10% formaldehyde for 10 minutes, washed again with distilled water and incubated with the Alizarin Red S staining solution for 5 minutes. After incubation the staining solution was removed and the cultures were washed 5 times with distilled water to get rid of excessive color.

For the *Oil Red O staining *a 0.5% Oil Red O stock solution in 2-Propanol was prepared. For the staining procedure the stock solution was diluted 3:2 with distilled water, 10 minutes incubated at room temperature and filtrated by using Whatmanpaper #1. The medium was removed; the cells were washed with PBS, fixed with 10% formalin for at minimum 30 minutes, and stained with the Oil Red O staining solution for one hour.

### Surface marker profiling by flow cytometry analysis

For flow cytometry analysis the cells were detached with 0.05% trypsin/EDTA and washed with PBS. 1 × 10^5 ^cells per tube were blocked for 5 minutes with human AB serum (Sigma) and stained for 15 minutes incubated in an ice bath in the dark with direct PE or FITC conjugated mouse anti-human monoclonal antibodies (Becton Dickinson) recognizing CD11b, CD34, CD38, CD44, CD45, CD73, CD90, CD117 and HLA-DR. After antibody incubation the cells were washed with PBS/sodium azide and resuspended in PBS in the needed volume for analyzing. 5 minutes before the analysis 7-AAD (0.25 μg per test) was applied to each sample for exclusion of dead cells. For negative control immunoglobulin isotype incubation was performed. FACS analyses were performed with FACS Calibur (Becton Dickinson).

### Sample preparation – RNA isolation

Total RNA was isolated with TRIzol reagent (GibcoBRL-Life Technologies) following the instructions of the manufacturer. RNA concentration was determined by photometric measurement. The RNA quality was analyzed by Agilent 2100 Bioanalyzer RNA assays and evaluated by calculating the ratio of the 28S and 18S ribosomal RNA intensity peaks.

### Microarray production

Human oligonucleotide microarrays were developed using 29550 oligonucleotides derived from 30 K MWG Human Oligo Set with 50 bp in length (provided by Prof. Reinhard Kofler, Tyrolian Cancer Research Institute), and dissolved in spotting buffer consisting of 3× SSC, 1.5 M Betaine. Microarrays were produced by spotting the reporters (including spotting buffer as negative control and human genomic DNA as positive control) in 48 blocks onto an epoxy-coated glass slide (Nexterion) resulting in 33456 features on a single high density array. Reporter molecules were fixed to the slides by baking at 42°C for 8 h at 50% relative humidity. RefSeq IDs indicate the genes represented by the oligonucleotides [see Additional file 3].

### Microarray hybridization procedure

The used labeling and hybridization procedures were based on those developed at The Institute for Genomic Research (TIGR) and modified at the Institute for Genomics and Bioinformatics [see Additional file 4 and 5]. All hybridizations were repeated with reversed dye assignment (dye-swap). After hybridization slides were scanned with GenePix 4000B microarray scanner (Axon Instruments) at 10 μm resolution. Identical settings were used for scanning the corresponding dye-swapped hybridized slides. The resulting TIFF images for each of the two fluorophores were analyzed with GenePix Pro 4.1 (Axon Instruments).

### Bioinformatics analyses

After image acquisition and filtering the data for low intensity, inhomogeneity and satured spots, the results files were normalized with the in-house developed software ArrayNorm [56]. After background correction the data sets were normalized by global-mean and dye-swap pairs or LOWESS-subgrid normalization. The obtained result files were used for further analyses such as cluster analysis [57], GO annotation [58] and pathway analyses [59]. Cluster analyses were performed with the Genesis software tool using *k*-means clustering function [28]. The number of clusters was varied from k = 1 to k = 15 and predictive power was analyzed with the figure of merit [60]. Subsequently, k = 12 was found optimal. GO annotation was performed on differentially expressed genes. Pathway analyses were performed with the in house developed Pathway Explorer software [59] which mapped the RefSeq IDs from the results file (cut-off level for differentially expressed genes: log2 ratio > 1) on public available pathways like Biocarta [61] and KEGG database [30]. All experimental parameters, images, raw and transformed data were uploaded to the microarray database MARS [62] and submitted via MAGE-ML export to a public repository (ArrayExpress, accession numbers A-MARS-2, E-MARS-3, E-MARS-4, E-MARS-5, and E-MARS-6) [63].

#### Real-time RT PCR

The same sources of total RNA used in the microarray experiments were used for the data validation by RT-PCR. The SuperScript™ II First Strand Synthesis System for RT PCR (Invitrogen) was used to synthesize cDNA of 5 μg RNA following manufacturers' recommendations. RNA contaminating genomic DNA was removed by treatment with DNAse I amplification grade (Invitrogen). The SuperScript™ II product was diluted 1:25 and directly used for the RT-PCR.

The RT-PCR assays were performed using the Eurogentec qPCR™ Mastermix Plus for Sybr™ Green I following manufacturer's recommendations. NO-reverse transcription Controls (No-RT) and No-Template Controls (NTC) were performed for each RNA type and primer pair. Cycling conditions on the ABI Prism 7000: 2 minutes at 50°C, 10 minutes at 95°C, 40 × (15 seconds at 95°C, 1 minute at 60°C) and the dissociation protocol at 60°C was added. For each sample RNA two cDNAs were prepared but in independent reverse transcription reactions. Each gene was tested with both cDNAs and the cDNAs were also tested in duplicate on one plate. Hence, four RT-PCR reactions were performed for the examination of one single gene. Further, the sample cDNA and the corresponding reference cDNA were tested on one plate, so that the data can be compared directly.

For normalization of the data the average of the expression level of four house-keeping genes, GAPDH, HPRT, beta-actin and beta-tubulin, was used.

## Authors' contributions

BK performed the experiments and analyzed the data. FSC was responsible for the microarray data normalization and statistical analysis. GF isolated the bone marrow aspirates. RW was responsible for the clinical studies. AJ and KHP supported the design of the experiments and the interpretation of the results. MS, CP, and AP developed and produced the microarrays. ZT was responsible for the overall conception and project coordination. All authors gave final approval of the version to be published.

## Additional files

There are 18 additional files provided with the online version of this manuscript comprising raw data and results of the analyses. The data files are available on our web site .

## Supplementary Material

Additional File 1 FACS analysis. Surface profiles in different passages. Click here for file

Additional File 2 Expression of stem cell specific marker genes evaluated by Realtime RT-PCR. Expression of stem cell specific marker genes evaluated by Realtime RT-PCR. Click here for file

Additional File 3 Array Design. Information on the spotted oligos. Click here for file

Additional File 4 SOP AMINOALLYL LABELING OF RNA FOR HUMAN OLIGO CHIPS. Standard Operating Procedure for AMINOALLYL LABELING OF RNA FOR HUMAN OLIGO CHIPS. Click here for file

Additional File 5 SOP HUMAN OLIGO CHIP PROBE HYBRIDIZATION. Standard Operating Procedure for microarray hybridization. Click here for file

Additional File 6 Differentially expressed ESTs. Differentially expressed ESTs (>2-foldchange) when comparing p2/p5 and p5/p10 for following moderated t-test Click here for file

Additional File 7 Validation of microarray data by Realtime RT-PCR. Gene expression analysis of long-term cultivated MSC. Click here for file

Additional File 8 Gene expression of osteoblast specific genes determined by Real-time RT-PCR. Gene expression of osteoblast specific genes determined by Real-time RT-PCR to prove the osteogenic phenotype. Click here for file

Additional File 9 Selected ESTs for microarray analysis. 1108 selected ESTs for microarray analysis. Click here for file

Additional File 10 k-means clustering. k-means clustering of 1108 selected ESTs. Click here for file

Additional File 11 Figure of merit analysis. Figure of merit analysis for validation of the k-value. Click here for file

Additional File 12 Hierarchical clustering. Hierarchical clustering of 1108 selected ESTs. Click here for file

Additional File 13 Validation of microarray data using real-time RT-PCR. Gene expression analysis of MSC during osteogenesis. Click here for file

Additional File 14 Functional annotation. Distribution of gene ontology (GO) terms in each cluster. Click here for file

Additional File 15 Expression matrix. Expression matrix of differentially expressed ESTs (>2-foldchange). Click here for file

Additional File 16 Selected ESTs for microarray analysis. 659 selected ESTs for microarray analysis. Click here for file

Additional File 17 Validation of microarray data by real-time RT-PCR. Real-time RT-PCR data. Click here for file

Additional File 18 Evaluation of human MSC as in vitro differentiation model. Comparison of human osteoblasts with in vitro differentiated hMSC. Click here for file
