# Supplementary material for: Gene expression profiling of human mesenchymal stem cells derived from bone marrow during expansion and osteoblast differentiation
Source: BMC Genomics. 2007 Mar 12;8:70. doi: 10.1186/1471-2164-8-70 (PMC1829400; doi:10.1186/1471-2164-8-70)
Supplement: Additional File 1 — FACS analysis. Surface profiles in different passages. [file 1471-2164-8-70-S1.pdf]

Overview of FACS histograms

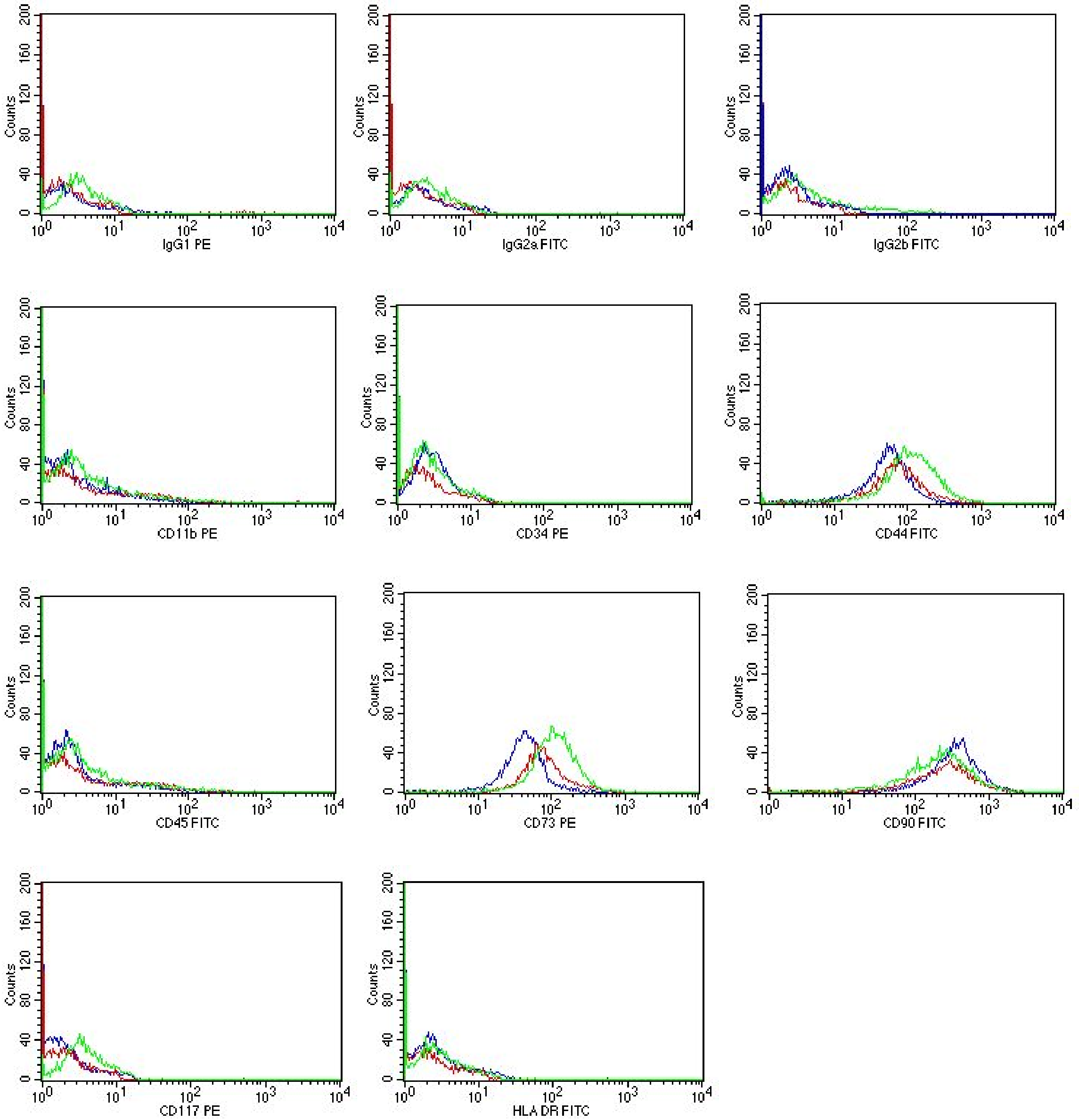

Overview of FACS histograms of the distinct surface marker (one representative donor shown); Histogram overlays of the three compared passages show that MSC do not change significantly their surface marker profile during long-term cultivation: CD11b-, CD34-, CD44+, CD45-, CD73+, CD90+, CD117-, and HLA-DR-; (blue line = passage 2; red line = passage 5; green line = passage 10)
