# Supplementary material for: Gene expression profiling of human mesenchymal stem cells derived from bone marrow during expansion and osteoblast differentiation
Source: BMC Genomics. 2007 Mar 12;8:70. doi: 10.1186/1471-2164-8-70 (PMC1829400; doi:10.1186/1471-2164-8-70)
Supplement: Additional File 2 — Expression of stem cell specific marker genes evaluated by Realtime RT-PCR. Expression of stem cell specific marker genes evaluated by Realtime RT-PCR. [file 1471-2164-8-70-S2.pdf]

Gene	Accessionnr.	Fold-change p2 vs. p5 (n=10)	Fold-change p10 vs. p5 (n=5)
CD 34	NM_001773	*Not detected*	*Not detected*
CD 44	NM_000610	0.91+/-0.10	1,29+/-0,36
CD 45	NM_080921	*Not detected*	*Not detected*
CD 73	NM_002526	0.89+/-0.08	1,37+/-0,32
CD 105	NM_000118	1.09+/-0.17	1,25+/-0,27
CD 166	NM_001627	1.3+/-0.16	0,95+/-0,24
CD 90	NM_006288	1.01+/-0.12	0,77+/-0,20
TERT	NM_003219	*Not detected*	*Not detected*
STRO1	NM_005862	1.15+/-0.16	1,10+/-0,28

Not detected means no band was visible on 2.5% agarose-gel with ethidium bromide

Gene expression of stem cell specific genes was determined by Realtime RT-PCR to examine if the expression of stem cell specific genes is changing during long-term cultivation. MSC from passage 2 were compared with MSC from passage 5 and MSC from passage 10 were compared with MSC from passage 5.
