# Supplementary material for: Gene expression profiling of human mesenchymal stem cells derived from bone marrow during expansion and osteoblast differentiation
Source: BMC Genomics. 2007 Mar 12;8:70. doi: 10.1186/1471-2164-8-70 (PMC1829400; doi:10.1186/1471-2164-8-70)
Supplement: Additional File 4 — SOP AMINOALLYL LABELING OF RNA FOR HUMAN OLIGO CHIPS. Standard Operating Procedure for AMINOALLYL LABELING OF RNA FOR HUMAN OLIGO CHIPS. [file 1471-2164-8-70-S4.pdf]

Standard Operating Procedure

TITLE: **AMINOALLYL LABELING OF RNA FOR HUMAN OLIGO CHIPS (HOC)**			
SOP-NO: MET014_01	BASED ON: TIGR SOP# M004		PAGE: 1 of 6
REWIEV PERIOD: biannual		VALID FROM: 01/01/2004	

originated:	Dipl.-Chem. Dr. M. Scheideler	the	date
approved by:	Dipl.-Ing. Dr.techn., Univ.- Prof. Z. Trajanoski	the	date

Contents

I .... PURPOSE

II ...... SCOPE

III ..... PROCEDURE

I. PURPOSE

This protocol describes the labeling of eukaryotic RNA with aminoallyl labeled nucleotides via first strand cDNA synthesis followed by a coupling of the aminoallyl groups to either Cyanine 3 or 5 (Cy 3/Cy5) fluorescent molecules.

II. SCOPE

This procedural format is currently utilized by Human Oligo Chip microarray projects under the supervision of Zlatko Trajanoski within the Bioinformatics Group, Biophysics Dept., Institute of Biomedical Engineering, Graz University of Technology, Graz Austria.

III. MATERIAL

3.1 5-(3-aminoallyl)-2'deoxyuridine-5'-triphosphate (AA-dUTP) (Sigma; Cat # A0410)
3.2 100 mM dNTP Set PCR grade (Life Technologies; Cat # 10297-018)
3.3 Random Hexamer primers (3mg/mL) (Life Technologies; Cat # 48190-011)
3.4 SuperScript II RT (200U/µL) (Life Technologies; Cat # 18064-014)
3.5 Cy-3 ester (AmershamPharmacia; Cat # PA23001)
3.6 Cy-5 ester (AmershamPharmacia; Cat # PA25001)
3.7 QIAquick PCR Purification Kit (Qiagen; Cat # 28106)
3.8 RNeasy® Mini Kit (Qiagen; Cat # 74106)

IV. REAGENT PREPARATION

4.1 Phosphate Buffers

4.1.1 Prepare 2 solutions: 1M K_2HPO4 and 1M KH_2PO4

4.1.2 To make a 1 M Phosphate buffer (KPO4, pH 8.5-8.7) combine:

1 M K_2HPO4........9.5 ml
1 M KH_2PO4........0.5 mL

4.1.3 For 100 mL Phosphate wash buffer (5 mM KPO4, pH 8.0, 80% EtOH) mix:

1 M KPO4 pH 8.5.... 0.5 mL
MilliQ water............ 15.25 mL
95% ethanol.......... 84.25 mL

Note: Wash buffer will be slightly cloudy.

4.1.4 Phosphate elution buffer is made by diluting 1 M KPO4, pH 8.5 to 4 mM with MilliQ water.

4.2 Aminoallyl dUTP

4.2.1 For a final concentration of 100 mM add 19.1 µL of 0.1 M KPO4 buffer (pH 7.5) to a stock vial containing 1 mg of aa-dUTP. Gently vortex to mix and transfer the aa-dUTP solution into a new microfuge tube. Store at –20 °C.

4.2.2 Measure the concentration of the aa-dUTP solution by diluting an aliquot 1:5000 in 0.1 M KPO4 (pH 7.5) and measuring the OD289. (Stock concentration in mM = OD289 x 704)

4.3 Labeling Mix (50x) with 2:3 aa-dUTP: dTTP ratio

4.3.1 Mix the following reagents:

		Final concentration
dATP (100 mM)	5µL	(25 mM)
dCTP (100 mM)	5µL	(25 mM)
dGTP (100 mM)	5µL	(25 mM)
dTTP (100 mM)	3µL	(15 mM)
aa-dUTP (100 mM)	2µL	(10 mM)
	Total: 20µL	

4.3.2 Store unused solution at –20 °C.

4.4 Sodium Carbonate Buffer (Na_2CO_3): 1 M, pH 9.0

4.4.1 Dissolve 10.8 g Na_2CO_3 in 80 mL of MilliQ water and adjust pH to 9.0 with 12 N HCl; bring volume up to 100 mL with MilliQ water.

4.4.2 To make a 0.1 M solution for the dye coupling reaction dilute 1:10 with water.

Note: Carbonate buffer changes composition over time; make it fresh every couple of weeks to a month.

4.5 Cy-dye esters

4.5.1 Cy3-ester and Cy5-ester are provided as a dried product in 5 tubes. Resuspend a tube of dye ester in 73 µL of DMSO before use.

4.5.2 Wrap all reaction tubes with foil and keep covered as much as possible in order to prevent photobleaching of the dyes.

Note: Dye esters must either be used immediately or aliquotted and stored at –80 °C. Any introduced water to the dye esters will result in a lower coupling efficiency due to the hydrolysis of the dye esters. Since DMSO is hygroscopic (absorbs water from the atmosphere) store it well sealed in desiccant.

V. PROCEDURE

5.1 Aminoallyl Labeling

5.1.1 To 10 µg of total RNA (or 2 µg poly(A+) RNA) which has been DNase I-treated and Qiagen RNeasy purified, add 2 µL Random Hexamer primers (3mg/mL) and bring the final volume up to 18.5 µL with RNase-free water.

5.1.2 Mix well and incubate at 70 °C for 10 minutes.

5.1.3 Snap-freeze in dry ice/ethanol bath for 30 seconds, centrifuge briefly at >10,000 rpm and continue at room temperature.

5.1.4 Add:

5x First Strand buffer............. 6 µL
0.1 M DTT............................... 3 µL
50x aminoallyl-dNTP mix......... 0.6 µL
SuperScript II RT (200 U/µL)... 2 µL

5.1.5 Mix and incubate at 42 °C for 3 hours to overnight.

5.1.6 To hydrolyze RNA, add:

1 M NaOH 10 µL
0.5 M EDTA 10 µL
mix and incubate at 65 °C for 15 minutes.

5.1.7 Add 10 µL of 1 M HCl to neutralize pH. (Alternatively, one can add 25 µL 1 M HEPES pH 7.0 or 25 µL 1 M Tris pH 7.4)

5.2 Reaction Purification I: Removal of unincorporated aa-dUTP and free amines (use the Qiagen method)

Qiagen Cleanup Method:

Note: This purification protocol is modified from the Qiagen QIAquick PCR purification kit protocol. The phosphate wash and elution buffers (prepared in 4.1.3 & 4.1.4) are substituted for the Qiagen supplied buffers because the Qiagen buffers contain free amines which compete with the Cy dye coupling reaction.

5.2.1 Mix cDNA reaction with 300 µL (5x reaction volume) buffer PB (Qiagen supplied) and transfer to QIAquick column.

5.2.2 Place the column in a 2 ml collection tube (Qiagen supplied) and centrifuge at ~13,000 rpm for 1 minute. Empty collection tube.

5.2.3 To wash, add 750 µL phosphate wash buffer to the column and centrifuge at ~13,000 rpm for 1 minute.

5.2.4 Empty the collection tube and repeat the wash and centrifugation step (5.2.3).

5.2.5 Empty the collection tube and centrifuge column an additional 1 minute at maximum speed.

5.2.6 Transfer column to a new 1.5 mL microfuge tube and carefully add 30 µL phosphate elution buffer (*see 4.1.4*) to the center of the column membrane.

5.2.7 Incubate for 1 minute at room temperature.

5.2.8 Elute by centrifugation at ~13,000 rpm for 1 minute.

5.2.9 Elute a second time into the same tube by repeating steps 5.2.6- 5.2.8. The final elution volume should be ~60 µL.

5.2.10 Dry sample in a speed vac with 45 minutes run time and 30 minutes medium heating time.

5.3 Coupling aa-cDNA to Cy Dye Ester

5.3.1 Resuspend aminoallyl-labeled cDNA in 4.5 µL 0.1 M sodium carbonate buffer (Na_2CO_3), pH 9.0.

Note: Carbonate buffer changes composition over time so make sure you make it fresh every couple of weeks to a month.

5.3.2 Add 4.5 µL of the appropriate NHS-ester Cy dye (prepared in DMSO: *see 4.5*)

Note: To prevent photobleaching of the Cy dyes wrap all reaction tubes in foil and keep them sequestered from light as much as possible.

5.3.3 Incubate the reaction for 1 hour in the dark at room temperature.

5.4 Reaction Purification II: Removal of uncoupled dye (Qiagen PCR Purification Kit)

5.4.1 To the reaction add 35 µL 100 mM NaOAc pH 5.2.

5.4.2 Add 250 µL (5x reaction volume) Buffer PB (Qiagen supplied).

5.4.3 Place a QIAquick spin column in a 2 mL collection tube (Qiagen supplied), apply the sample to the column, and centrifuge at ~13,000 for 1 minute. Empty collection tube.

5.4.4 To wash, add 750 µL Buffer PE (Qiagen supplied) to the column and centrifuge at ~13,000 for 1 minute.

Note: Make sure Buffer PE has added ethanol before using (see label for correct volume).

5.4.5 Empty collection tube and centrifuge column for an additional 1 minute at maximum speed. The column bottom should have the colour of the incorporated dye.

5.4.6 Place column in a clean 1.5 mL microfuge tube and carefully add 30 µL Buffer EB (Qiagen supplied) to the center of the column membrane.

5.4.7 Incubate for 1 minute at room temperature.

5.4.8 Elute by centrifugation at ~13,000 rpm for 1 minute.

5.4.9 Elute a second time into the same tube by repeating steps 5.4.6- 5.4.8. The final elution volume should be ~60 µL. The solution should have the colour of the incorporated dye.

Note: This protocol is modified from the Qiagen QIAquick Spin Handbook (04/2000, pg. 18).

5.5 Analysis of Labeling Reaction (optional)

5.5.1 Use a 50 µL Beckman quartz MicroCuvette to analyze the entire und diluted sample in a spectrophotometer.

5.5.2 Wash the cuvette with water and blow dry with compressed air duster.

5.5.3 Pipette sample into cuvette and place cuvette in spectrophotometer.

5.5.4 For each sample measure absorbance at 260 nm and either 550 nm for Cy3 or 650 nm for Cy5, as appropriate.

5.5.5 Pipette sample from cuvette back into the original sample tube.

5.5.6 For each sample calculate the total picomoles of cDNA synthesized using:

$$\text{pmol nucleotides} = \frac{\text{[OD260 * volume (µL) * 37 ng/µL * 1000 pg/ng]}}{\text{324.5 pg/pmol}}$$

Note: 1 OD260 = 37 ng/µL for cDNA; 324.5 pg/pmol average molecular weight of a dNTP)

5.5.7 For each sample calculate the total picomoles of dye incorporation (Cy3 or Cy5 accordingly) using:

$$\text{pmol Cy3} = \frac{\text{OD550 * volume (µL)}}{0.15}$$

$$\text{pmol Cy5} = \frac{\text{OD650 * volume (µL)}}{0.25}$$

$$\text{nucleotides/dye ratio} = \frac{\text{pmol cDNA}}{\text{pmol Cy dye}}$$

Note: >200 pmol of dye incorporation per sample and a ratio of less than 50 nucleotides/dye molecules is optimal for hybridizations (see Microarray Cookbook II)

5.6 Drying Slides

5.6.1 Dry the Cy3/Cy5 probe mixture to completion in a speed vac and continue with SOP: Met015 for the hybridization of the probe to a microarray slide.
