# Supplementary material for: Gene expression profiling of human mesenchymal stem cells derived from bone marrow during expansion and osteoblast differentiation
Source: BMC Genomics. 2007 Mar 12;8:70. doi: 10.1186/1471-2164-8-70 (PMC1829400; doi:10.1186/1471-2164-8-70)
Supplement: Additional File 5 — SOP HUMAN OLIGO CHIP PROBE HYBRIDIZATION. Standard Operating Procedure for microarray hybridization. [file 1471-2164-8-70-S5.pdf]

Standard Operating Procedure

TITLE: **HUMAN OLIGO CHIP (HOC) PROBE HYBRIDIZATION**		
SOP-NO: MET015_00	BASED ON: TIGR SOP# M005	PAGE: 1 of 5
REWIEV PERIOD: biannual	VALID FROM: 21/08/2003	

originated: ______________________ the ______________________
Dipl.-Chem. Dr. M. Scheideler — date

approved by: ______________________ the ______________________
Dipl.-Ing. Dr.techn., Univ.- Prof. Z. Trajanoski — date

Contents

I .... PURPOSE

II ...... SCOPE

III ..... PROCEDURE

IV ..... ENCLOSURE

I. PURPOSE

This protocol describes the hybridization of a Cy labeled cDNA probe (mix of Cy3 and Cy5) onto epoxy coated slide spotted with 50mer oligonucleotides.

II. SCOPE

This procedural format is currently utilized by Human Oligo Chip microarray projects under the supervision of Zlatko Trajanoski within the Bioinformatics Group, Biophysics Dept., Institute of Biomedical Engineering, Graz University of Technology, Graz Austria.

III. MATERIAL

3.1 20x Saline-Sodium Citrate (SSC) (Sigma; Cat # S-6639)
3.2 10% Sodium Dodecyl Sulfate (SDS)(Life Technologies; Cat # 15553-035)
3.3 Bovine Serum Albumin (BSA) (Sigma; Cat # A-9418)
3.4 Formamide, redistilled (Life Technologies; Cat # 15515-081)
3.5 Isopropanol (Fisher Scientific; Cat # A451-1)
3.6 Coplin jar (VWR; Cat # 25457-200)
3.7 Human COT1-DNA (Life Technologies; Cat # 15279-011)
3.8 Mouse COT1-DNA (Life Technologies; Cat # 18440-016)
3.9 Poly(A)-DNA (Pharmacia; Cat # 27-7836-01)
3.10 Microscope Cover Glass (Fisher Scientific; Cat # 12-545J)
3.11 Human Oligo Chips produced by spotting 50mer oligonucleotides (MWG, Ebersberg) onto epoxy coated glass slides (Quantifoil) at the Bioinformatics Group, Biophysics Dept., Institute of Biomedical Engineering, Graz University of Technology, Graz Austria
3.12 Hybridization chamber (Corning Costar; Cat #2551)
3.13 1 L .22 µm CA (cellulose acetate) Filter System (Corning; Cat #430517)
3.14 Pressurized air duster (Fellowes; Cat # 99790) or clean in-house pressurized air source

IV. PROCEDURE

4.1 Capture Probe Fixation

4.1.1 Epoxy coated slides (Quantifoil) spotted with 50mer oligonucleotides in 3x SSC, 1.5 M Betaine are baked at 42 °C for 8 hours at 50% rel. humidity.

4.2 Prehybridizaton

4.2.1 Prepare prehybridization buffer (5x SSC, 0.1% SDS, 1% BSA) and sterilize by filtration using a CA filter. Preheat at 42 °C for ~30 minutes before use.

4.2.2 Place the printed slide(s) which will be used for the hybridization in a Coplin jar containing preheated prehybridization buffer and incubate at 42 °C for 45 minutes.

4.2.3 Washing Slides

- Fill five Coplin jars with MilliQ water and another with isopropanol.
- With forceps carefully grasp slide by the labeled end and vertically dip slide into the first Coplin jar (water) so that the slide is completely submerged. Dip slide five times.
- Dip the slide again in the water five times but only submerging the slide enough to wash the printed array itself.
- Using the same technique dip slide into the following Coplin jars (water).
- Finally dip the slide into the sixth Coplin jar (isopropanol) submerging the slide completely.

Note: Replace each water wash after every five slides.

4.2.4 Dry slides by immediate centrifugation at 1500 rpm for 2 minutes

Note:
- Note the general appearance of the slide. Streaking or mottling on the slide surface indicates further washing is necessary.
- Repeat the water/water/isopropanol wash cycle as necessary to clean the slide. Blow dry between each cycle.
- When working with an oligonucleotide array one can denature the spotted oligos by submerging the slide in a 95 °C MilliQ water bath for 3 minutes just after prehybridization and before adding the probe.

4.2.5 Use slides immediately following prehybridization to ensure optimal hybridization efficiency.

4.3 Hybridization

4.3.1 Prepare 1x hybridization buffer (50% formamide, 5X SSC, and 0.1% SDS).

4.3.2 Prepare Poly(A)-DNA by dissolving stock Poly(A)-DNA in a neutral buffer (i.e. 10 mM Tris, pH 7) to a final concentration of 20 µg/µL.

4.3.3 Prepare COT1-DNA (stock conc.1µg/µL) by ethanol precipitation:

- Add 2 to 3 volumes of ethanol and 0.1 volumes of 3 M Sodium Acetate (NaOAc) to the stock tube.
- Mix well and place on dry ice for 20-30 minutes or in –20 °C freezer overnight.
- Centrifuge for 20-30 minutes in a cold room microfuge at maximum angular velocity.
- Remove supernatant and allow excess ethanol to dry off.
- Dissolve precipitated COT1 in a neutral buffer (i.e. 10 mM Tris, pH 7) to the final concentration of 20 µg/µL.

4.3.4 Resuspend labeled probe (Cy3/Cy5 probe mixture: *see SOP-Met014*) in 24 µL of 1x hybridization buffer.

Note: Expose Cy labeled probe to light as little as possible during the hybridization process.

4.3.5 To block nonspecific hybridization add:

COT1-DNA (20 µg/µL)……. 1µL
Poly(A)-DNA (20µg/µL)…... 1µL

Note: The COT1–DNA is organism specific: add mouse COT1 to labeled mouse probes and human COT1-DNA to labeled human probes.

4.3.6 To denature, heat the probe mixture at 95 °C for 3 minutes and snap cool on ice for 30 sec.

4.3.7 Centrifuge the probe mixture at maximum angular velocity for 1 minute. Keep at room temperature and use immediately.

4.3.8 To Apply Labeled Probe Mixture

- Place a prehybridized microarray slide (array side up) between the guide teeth in the bottom half of a hybridization chamber.
- Pipette the labeled probe mixture (~26 µL) to the slide surface near one end of the array print area keeping bubbles to a minimum.
- Take a 22mm x 60mm microscope glass coverslip, dust it with compressed air, and grasp one end with forceps.
- Holding the coverslip over the array print area, lower the end nearest the pool of cDNA probe until solution wicks to the surface of the coverslip.
- Gradually lower the opposite end of the coverslip (held by the forceps) onto the slide. The solution may take a minute or two to wick across the entire length of the slide.
- After probe has wicked across the slide carefully adjust the coverslip's position with the tip of the forceps so that there is an even margin between the edge of the coverslip and the edge of the slide.
- Work any large bubbles toward the edge by gently tapping the coverslip surface; small bubbles will absolve themselves during hybridization.

4.3.9 To the small wells at each end of the chamber add 10 µL of water (20µL total), cover, and seal the chamber.

4.3.10 Wrap the chamber in foil (light-tight) and incubate in a 42 °C water bath for 16-20 hours. To ensure chamber remains level and does not float to the surface place a small weight upon it.

Note: Do not flip the hybridization chamber upside down during hybridization; this may cause the coverslip to shift from the slide and adversely affect the hybridization.

4.4 Washing Slides after hybridization

4.4.1 Prepare a low stringency wash buffer I (~500mL) containing 2x SSC and 0.1% SDS

4.4.2 Prepare a low stringency wash buffer II (~500mL) containing 1x SSC

4.4.3 Prepare a high-stringency wash buffer III (~500mL) containing 0.5x SSC.

4.4.4 Preheat all wash buffers (I-III) to 30 °C before usage. Fill one Coplin jar with wash buffer I and preheat to 42 °C before usage.

4.4.5 After the incubation remove foil and unseal hybridization chamber. Remove the slide from the chamber, taking care not to disturb the coverslip.

4.4.6 To remove coverslip submerge slide in a the Coplin jar containing low stringency wash buffer I (preheated to 42 °C) for 2 minutes. With time the coverslip will slide free of the slide surface.

Note: Once the slide has been hybridized it should be exposed to light as little as possible. Therefore, all staining dishes should be covered with foil to make them light tight.

4.4.7 After the coverslip is removed place slide in a staining dish containing low stringency wash buffer I (2x SSC and 0.1% SDS, preheated to 30 °C) and agitate for 5 minutes at room temperature.

4.4.8 Wash the slide in a staining dish with low stringency wash buffer II (1x SSC, preheated to 30 °C) by agitating for 5 minutes at room temperature.

4.4.9 Wash the slide in high-stringency wash buffer III (0.5x SSC, preheated to 30 °C) agitating for 5 minutes at room temperature.

4.4.10 Finally dry the slides by immediate centrifugation at 1500 rpm for 2 minutes using the same technique as step 4.2.4.

4.4.11 Place slides in a light tight slide box until they can be scanned, preferably as soon as possible.

V. ENCLOSURE

No enclosure given.
