# Supplementary material for: Gene expression profiling of human mesenchymal stem cells derived from bone marrow during expansion and osteoblast differentiation
Source: BMC Genomics. 2007 Mar 12;8:70. doi: 10.1186/1471-2164-8-70 (PMC1829400; doi:10.1186/1471-2164-8-70)
Supplement: Additional File 7 — Validation of microarray data by Realtime RT-PCR. Gene expression analysis of long-term cultivated MSC. [file 1471-2164-8-70-S7.pdf]

Validation of Microarray data by independent Realtime RT-PCR

Gene expression analysis of long-term cultivated hMSC

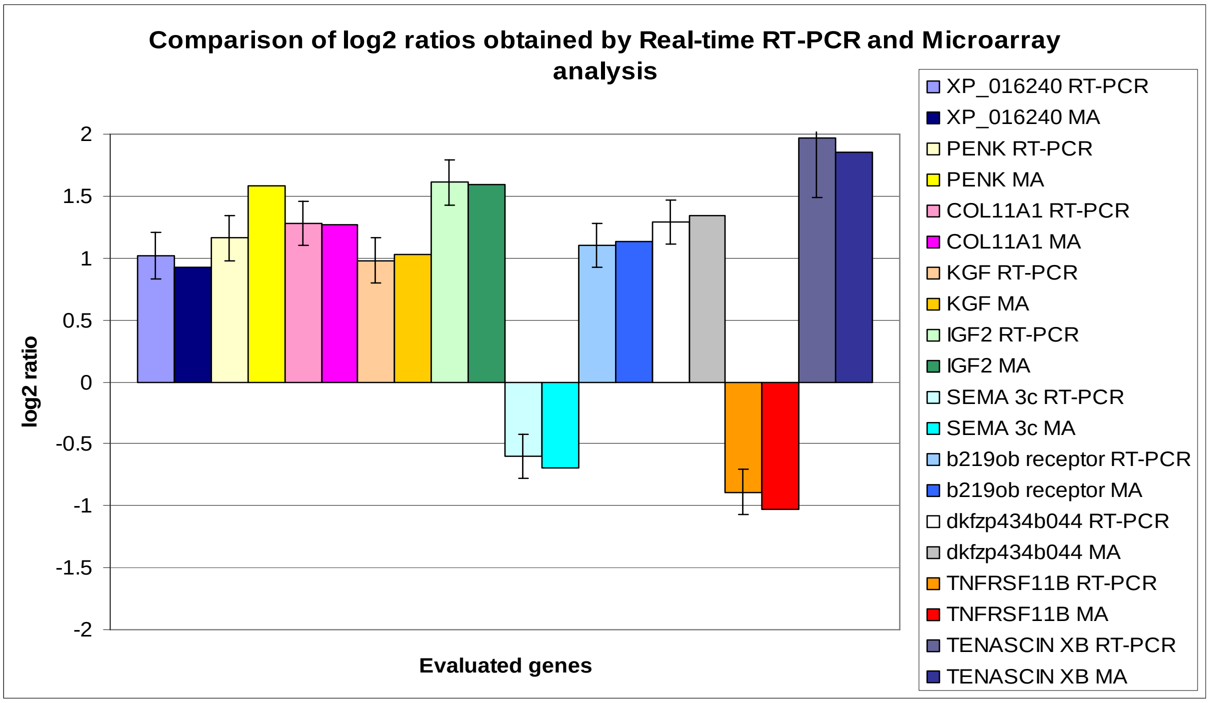

Comparison of log2-ratios obtained by Real-time-RT PCR and microarray analysis for confirming the data set obtained by microarray analysis.
