# Supplementary material for: Gene expression profiling of human mesenchymal stem cells derived from bone marrow during expansion and osteoblast differentiation
Source: BMC Genomics. 2007 Mar 12;8:70. doi: 10.1186/1471-2164-8-70 (PMC1829400; doi:10.1186/1471-2164-8-70)
Supplement: Additional File 8 — Gene expression of osteoblast specific genes determined by Real-time RT-PCR. Gene expression of osteoblast specific genes determined by Real-time RT-PCR to prove the osteogenic phenotype. [file 1471-2164-8-70-S8.pdf]

Acc.No.	Gene name	MSCd-1/MSC d4	MSC d-1/MSC d7	MSC d-1/MSC d14	MSC d-1/ MSC d21
NM_000478	ALPL	0.97	1.34	1.02	0.27
NM_002449	MSX2	1.09	1.11	1.85	1.92
NM_000376	VDR	-0,34	-0,23	0,48	0,88
NM_000088	COL1A1	0.24	0.60	0.39	2.54
NM_003118	SPARC	1.17	0.71	1.43	1.71
NM_000582	SPP1	1.66	0.53	1.66	1.53
NM_007221	BGLAP	1.17	0.71	1.43	1.71
	Stdev.	+/- 0.09	+/- 0.09	+/- 0.14	+/- 0.26

Gene expression of osteoblast specific genes was determined by Real-time RT-PCR to prove the osteogenic phenotype. The table shows the log2 ratios and the standard deviation when comparing undifferentiated MSC (MSCd-1) with MSC on distinct days after osteogenic induction (MSC d4, d7, d14, d21).
