# Supplementary material for: Gene expression profiling of human mesenchymal stem cells derived from bone marrow during expansion and osteoblast differentiation
Source: BMC Genomics. 2007 Mar 12;8:70. doi: 10.1186/1471-2164-8-70 (PMC1829400; doi:10.1186/1471-2164-8-70)
Supplement: Additional File 10 — k-means clustering. k-means clustering of 1108 selected ESTs. [file 1471-2164-8-70-S10.pdf]

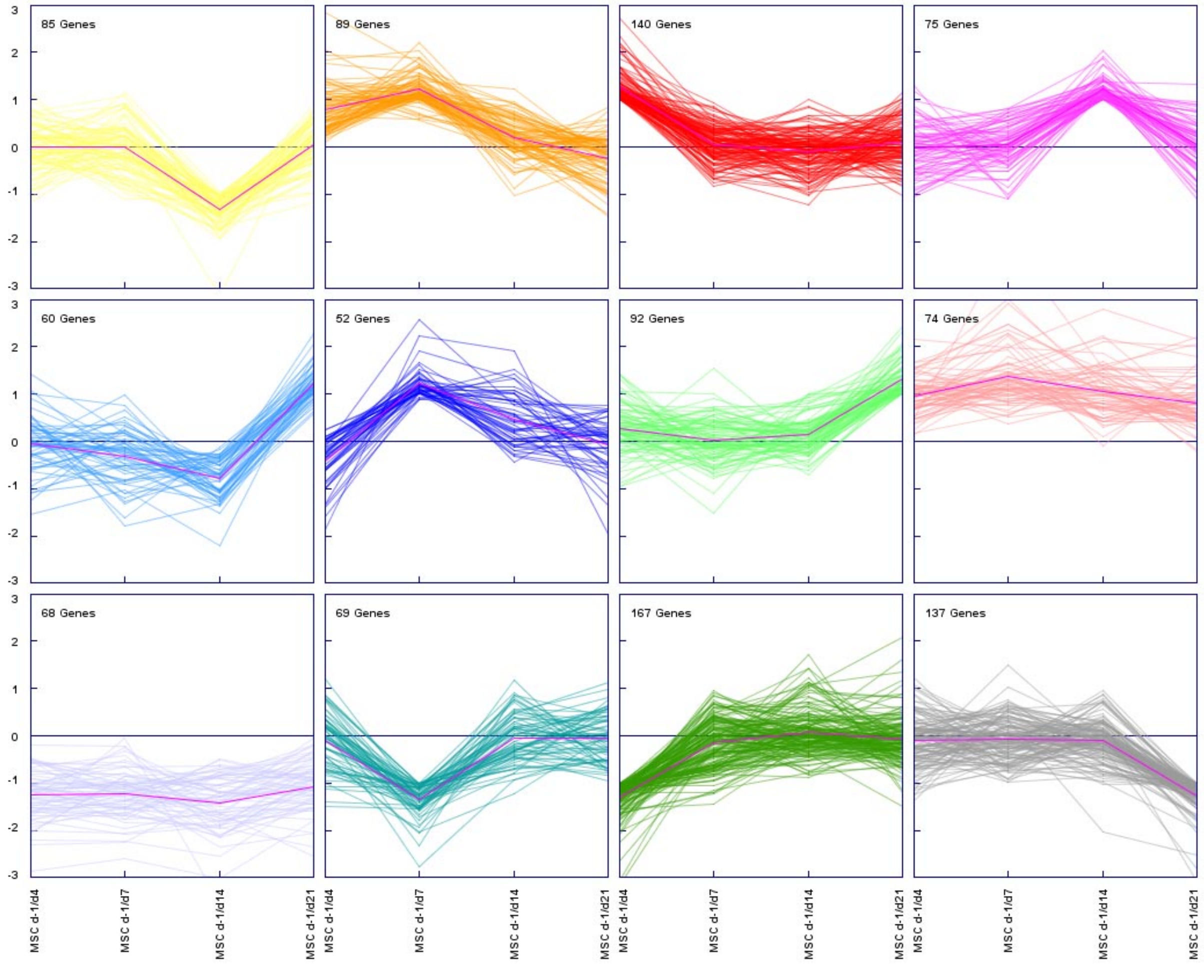

Expression view of all by *k*-means clustering calculated clusters. *K*-means clustering was performed for 1108 selected genes/ESTs shown to be more than two fold up or down regulated at at least one time point during osteogenic differentiation. Genes were grouped in 12 clusters with distinct expression profiles. Relative expression levels (log2 ratios) are shown for each gene at different time point.
