# Supplementary figures and images for: Gene expression profiling of human mesenchymal stem cells derived from bone marrow during expansion and osteoblast differentiation
Source: BMC Genomics. 2007 Mar 12;8:70. doi: 10.1186/1471-2164-8-70 (PMC1829400; doi:10.1186/1471-2164-8-70)

FOM Value versus Number of Clusters

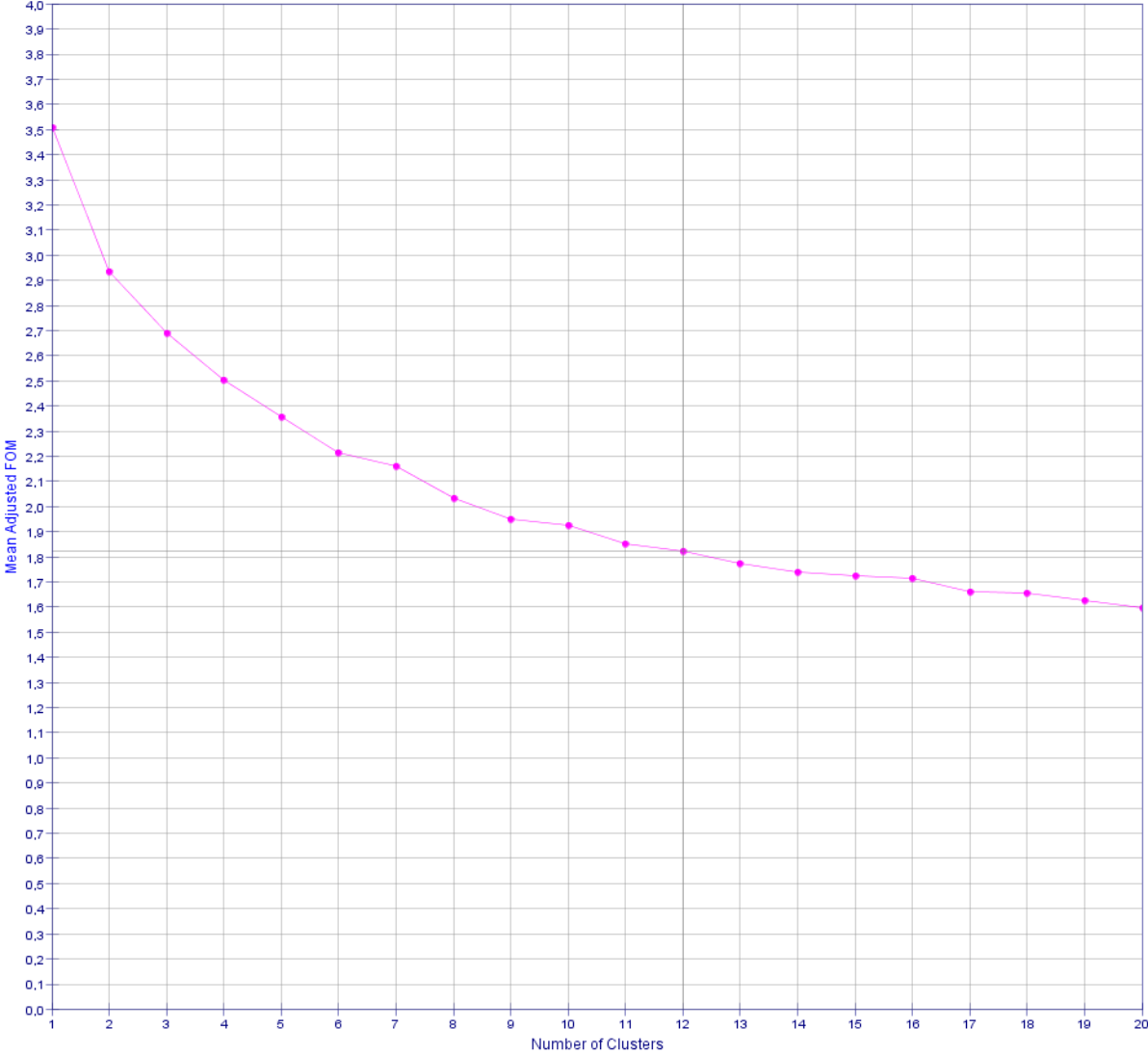

Supplement: Additional File 11 — Figure of merit analysis. Figure of merit analysis for validation of the k-value. [file 1471-2164-8-70-S11.pdf]

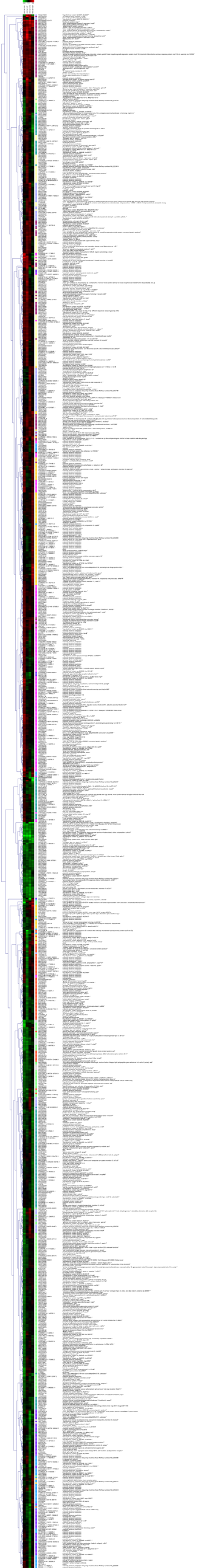

Supplement: Additional File 12 — Hierarchical clustering. Hierarchical clustering of 1108 selected ESTs. [file 1471-2164-8-70-S12.png]

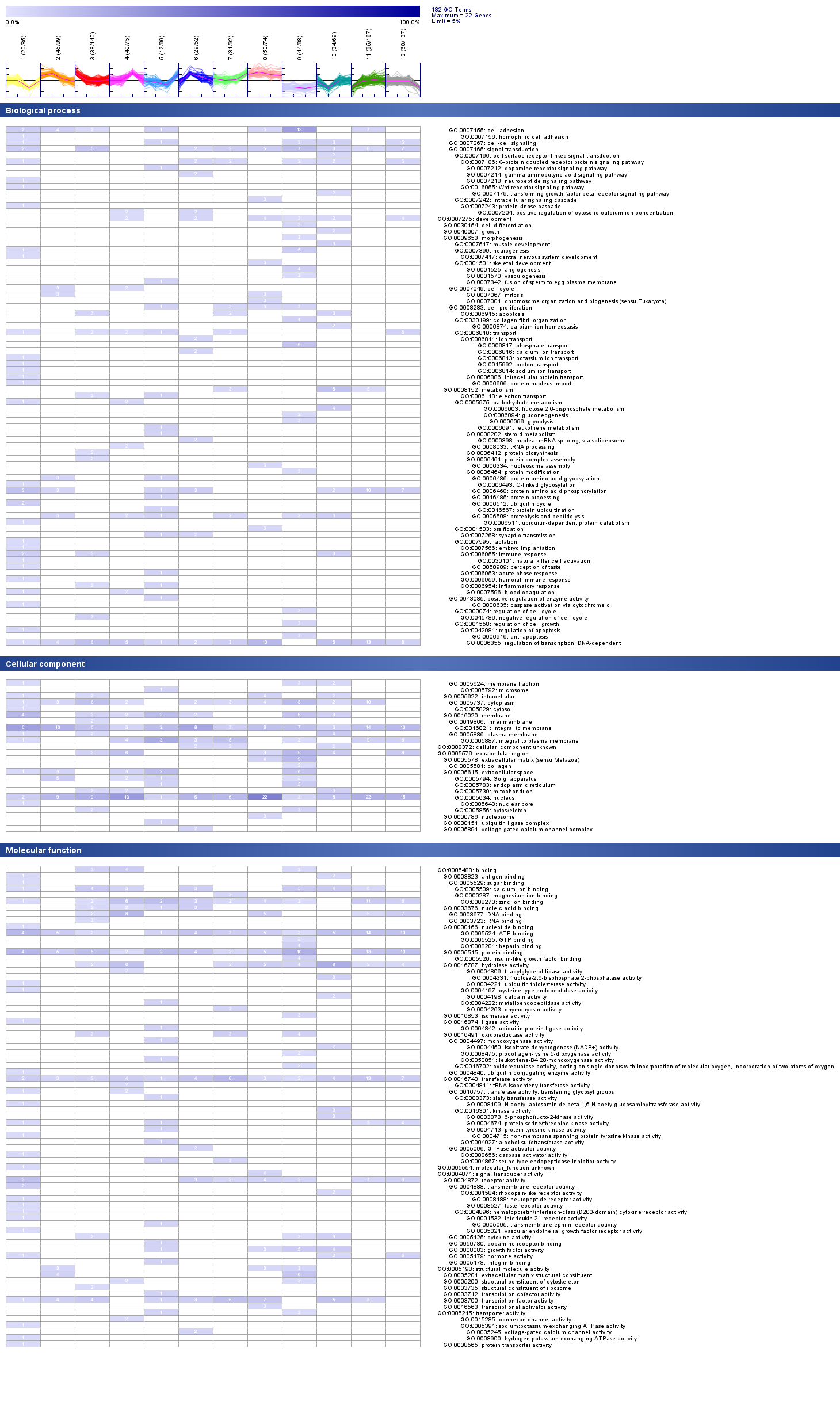

Supplement: Additional File 14 — Functional annotation. Distribution of gene ontology (GO) terms in each cluster. [file 1471-2164-8-70-S14.png]

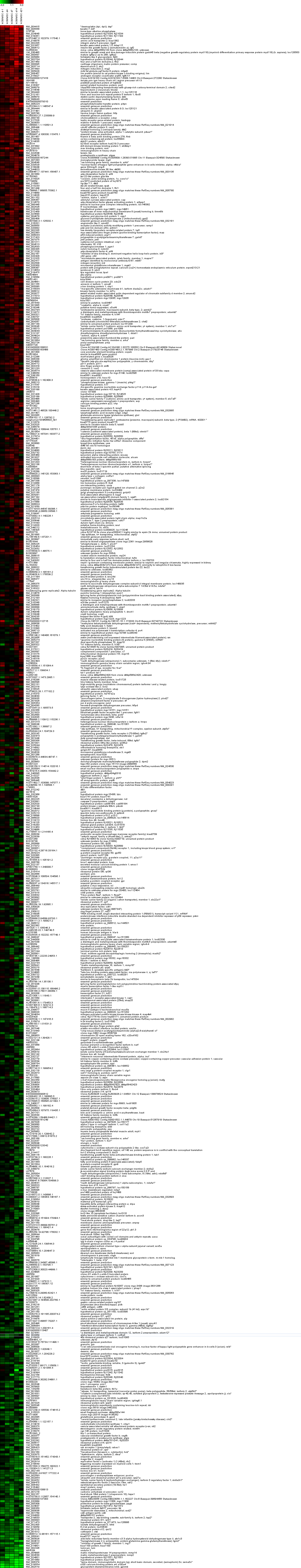

Supplement: Additional File 15 — Expression matrix. Expression matrix of differentially expressed ESTs (>2-foldchange). [file 1471-2164-8-70-S15.png]
