# Supplementary material for: Gene expression profiling of human mesenchymal stem cells derived from bone marrow during expansion and osteoblast differentiation
Source: BMC Genomics. 2007 Mar 12;8:70. doi: 10.1186/1471-2164-8-70 (PMC1829400; doi:10.1186/1471-2164-8-70)
Supplement: Additional File 17 — Validation of microarray data by real-time RT-PCR. Real-time RT-PCR data. [file 1471-2164-8-70-S17.pdf]

Validation of Microarray data by independent Realtime RT-PCR

Gene expression analysis of hOB compared with hMSC during osteogenesis

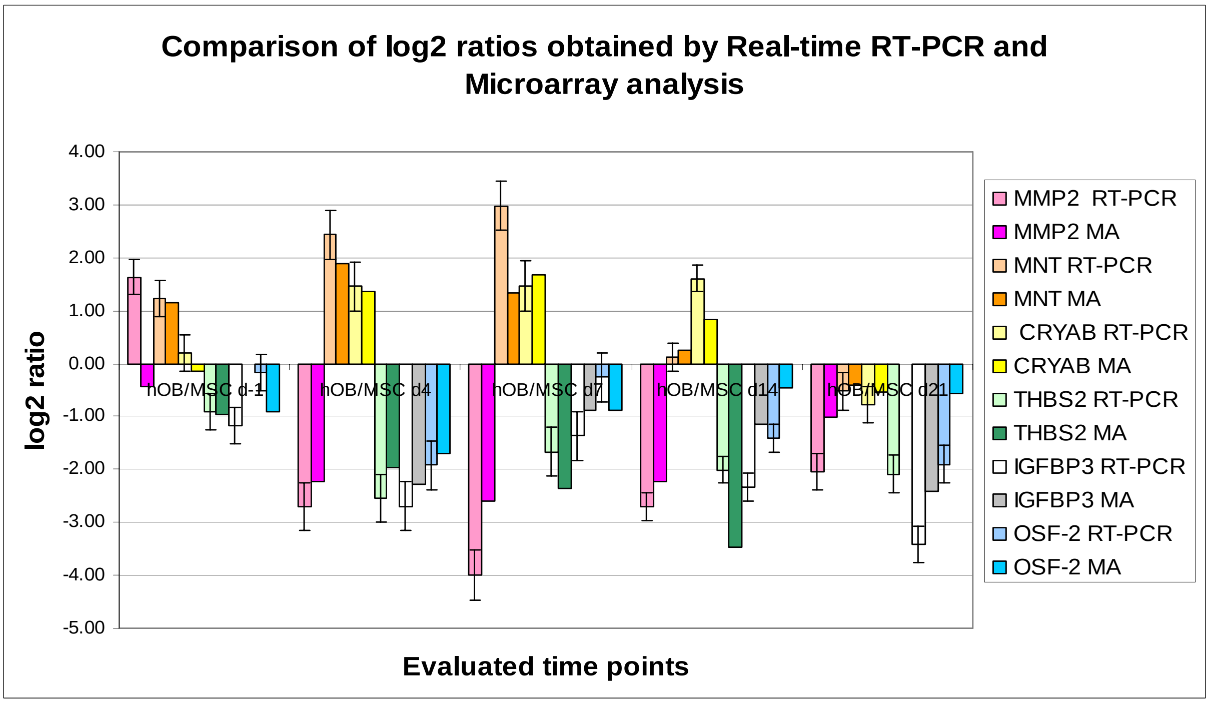

Comparison of log2 ratios obtained by Real-time RT-PCR and microarray analysis for confirming the microarray data set.
