# Supplementary material for: Gene expression profiling of human mesenchymal stem cells derived from bone marrow during expansion and osteoblast differentiation
Source: BMC Genomics. 2007 Mar 12;8:70. doi: 10.1186/1471-2164-8-70 (PMC1829400; doi:10.1186/1471-2164-8-70)
Supplement: Additional File 18 — Evaluation of human MSC as in vitro differentiation model. Comparison of human osteoblasts with in vitro differentiated hMSC. [file 1471-2164-8-70-S18.pdf]

Overview of differentially expressed genes in human osteoblasts (OB) compared with MSC differentiating into osteoblasts.

	**Differentially expressed genes with cut-off level 2 fold change (log1)**	**Percentage of total analysed genes (28606)**
OB/MSC day 1	614 (238 up / 376 down)	2.14%
OB/MSC day 4	521 (203 up / 322 down)	1.82%
OB/MSC day 7	399 (223 up / 176 down)	1.39%
OB/MSC day 14	757 (377 up / 381 down)	2.65%
OB/MSC day 21	537 (272 up / 311 down)	1.88%
